# Evodiamine Inhibits Insulin-Stimulated mTOR-S6K Activation and IRS1 Serine Phosphorylation in Adipocytes and Improves Glucose Tolerance in Obese/Diabetic Mice

**DOI:** 10.1371/journal.pone.0083264

**Published:** 2013-12-31

**Authors:** Ting Wang, Tatsuya Kusudo, Tamaki Takeuchi, Yukari Yamashita, Yasuhide Kontani, Yuko Okamatsu, Masayuki Saito, Nozomu Mori, Hitoshi Yamashita

**Affiliations:** 1 Department of Biomedical Sciences, College of Life and Health Sciences, Chubu University, Kasugai, Japan; 2 Nutritional Health Science Research Center, Chubu University, Kasugai, Japan; 3 Department of Food Science for Health, Minami-Kyushu University, Miyazaki, Japan; 4 Department of Biomedical Sciences, Graduate School of Veterinary Medicine, Hokkaido University, Sapporo, Japan; 5 Department of Nutrition, School of Nursing and Nutrition, Tenshi College, Sapporo, Japan; 6 Department of Anatomy and Neurobiology, Nagasaki University School of Medicine, Nagasaki, Japan; Pennington Biomedical Research Center, United States of America

## Abstract

Evodiamine, an alkaloid extracted from the dried unripe fruit of the tree *Evodia rutaecarpa* Bentham (Rutaceae), reduces obesity and insulin resistance in obese/diabetic mice; however, the mechanism underlying the effect of evodiamine on insulin resistance is unknown. This study investigated the effect of evodiamine on signal transduction relating to insulin resistance using obese/diabetic KK-Ay mice and an *in vitro* adipocyte culture. There is a significant decrease in the mammalian target of rapamycin (mTOR) and ribosomal S6 protein kinase (S6K) signaling in white adipose tissue (WAT) in KK-Ay mice treated with evodiamine, in which glucose tolerance is improved. In addition, reduction of insulin receptor substrate 1 (IRS1) serine phosphorylation, an indicator of insulin resistance, was detected in their WAT, suggesting suppression of the negative feedback loop from S6K to IRS1. As well as the stimulation of IRS1 and Akt serine phosphorylation, insulin-stimulated phosphorylation of mTOR and S6K is time-dependent in 3T3-L1 adipocytes, whereas evodiamine does not affect their phosphorylation except for an inhibitory effect on mTOR phosphorylation. Moreover, evodiamine inhibits the insulin-stimulated phosphorylation of mTOR and S6K, leading to down-regulation of IRS1 serine phosphorylation in the adipocytes. Evodiamine also stimulates phosphorylation of AMP-activated protein kinase (AMPK), an important regulator of energy metabolism, which may cause down-regulation of mTOR signaling in adipocytes. A similar effect on AMPK, mTOR and IRS1 phosphorylation was found in adipocytes treated with rosiglitazone. These results suggest evodiamine improves glucose tolerance and prevents the progress of insulin resistance associated with obese/diabetic states, at least in part, through inhibition of mTOR-S6K signaling and IRS1 serine phosphorylation in adipocytes.

## Introduction

The increased availability of food in Western countries and in Japan has augmented the prevalence of obesity and insulin resistance, which are central in the development of metabolic syndrome [1]. High fat intake is considered to be the major cause of metabolic abnormalities due to overnutrition. Adipose tissue is responsible for the majority of fat metabolism affecting largely glucose metabolism and insulin sensitivity under the control of various hormones and cytokines [2]. Nutrient overload increases serum insulin levels, which stimulates uptake of free fatty acids (FFAs) and glucose into adipocytes mainly in white adipose tissue (WAT), where excess energy is stored in the form of triglycerides. When this energy storage system is active, increases in the number and size of adipocytes are required for additional fat deposition in WAT, causing excessive growth of adipose tissue leading to obesity [3]. Elevated levels of serum insulin as well as non-esterified FFAs in the obese state decrease insulin sensitivity and increase insulin resistance in metabolic tissues, including liver, muscle and adipose tissue, leading to type 2 diabetes mellitus. Regulation of WAT is a potential strategy for the treatment of obesity and for improvement of insulin resistance. Brown adipose tissue (BAT) is specialized for thermogenesis through the function of uncoupling protein 1 (UCP1) located in the mitochondria [4], [5]. Because of UCP1, which dissipates caloric energy as heat, BAT has an important role in preventing obesity, as shown in our earlier study using UCP1-knockout mice [6]. It was found recently that functional BAT, despite its reduction with age, exists in adult humans and its level is correlated inversely to the degree of adiposity [7], [8]. These findings have accelerated basic and clinical studies on the stimulation of BAT formation and activity as a potential therapeutic target against obesity and insulin resistance [9]; however, an alternative strategy independent of UCP1 thermogenesis is needed for BAT-negative individuals.

Insulin signaling is implicated in the regulation of adipocyte biology. Many of the metabolic and anti-apoptotic effects of insulin are mediated by the signaling pathway beginning from phosphorylation and activation of insulin/insulin-like growth factor I receptors, which results in tyrosine phosphorylation of the insulin receptor substrate 1 (IRS1) [10]. Activation of phosphatidylinositol 3-kinase (PI3K) and protein kinase B/Akt by the IRS1 protein appears to be important in the mechanism of glucose uptake in adipocytes and muscle cells. In addition, insulin signaling is intimately linked to the nutrient-responsive mammalian target of rapamycin (mTOR) signaling pathway via activation of Akt [11], [12]. The activation of mTOR phosphorylates its downstream protein ribosomal S6 protein kinase (S6K), participating in several processes including protein synthesis and proliferation [12], [13]. It became apparent that serine phosphorylation of IRS1 reduces the ability of IRS1 to activate PI3K [10]–[12]. In diet-induced obesity, overactivation of mTOR-S6K signaling favors expansion of the WAT mass, leading to insulin resistance of adipocytes through elevated serine phosphorylation of IRS1. Mice deficient of S6K are protected against diet-induced obesity and show enhanced insulin sensitivity owing to the loss of the negative feedback loop from S6K to IRS1 [14], [15]. These findings suggest further development of interventions targeting mTOR-S6K signaling for the treatment and prevention of obesity and insulin resistance.

Evodiamine, an alkaloid extracted from the dried unripe fruit of *Evodia rutaecarpa* Bentham (Rutaceae), has been used for many years as a traditional Chinese herbal medicine for the treatment of pain, vomiting and pyresis. Evodiamine has a wide variety of bioactivity with antinociceptive, anti-obesity, vasodilatory, anti-tumor and anti-inflammatory effects [16]–[20]. We found evodiamine decreases diet-induced obesity and glucose intolerance in a UCP1-independent manner in mice [21]. We showed that evodiamine increases phosphorylation of extracellular signal-regulated kinase (ERK) and reduces the expression of transcription factors such as PPARγ in pre-adipocytes, strongly inhibiting their differentiation into mature adipocytes. It was shown recently that evodiamine improves insulin resistance and fat accumulation in obese/diabetic db/db mice [22]. However, the mechanisms underlying the effects of evodiamine on glucose tolerance and insulin resistance are not known. This study used obese/diabetic mice and adipocyte culture *in vitro* to investigate how evodiamine affects glucose tolerance and insulin resistance, especially from the viewpoint of signal transduction, including the mTOR-S6K signaling pathway.

## Results

### Effects of evodiamine on metabolic phenotypes in obese/diabetic KK-Ay mice

**Figure 1:**
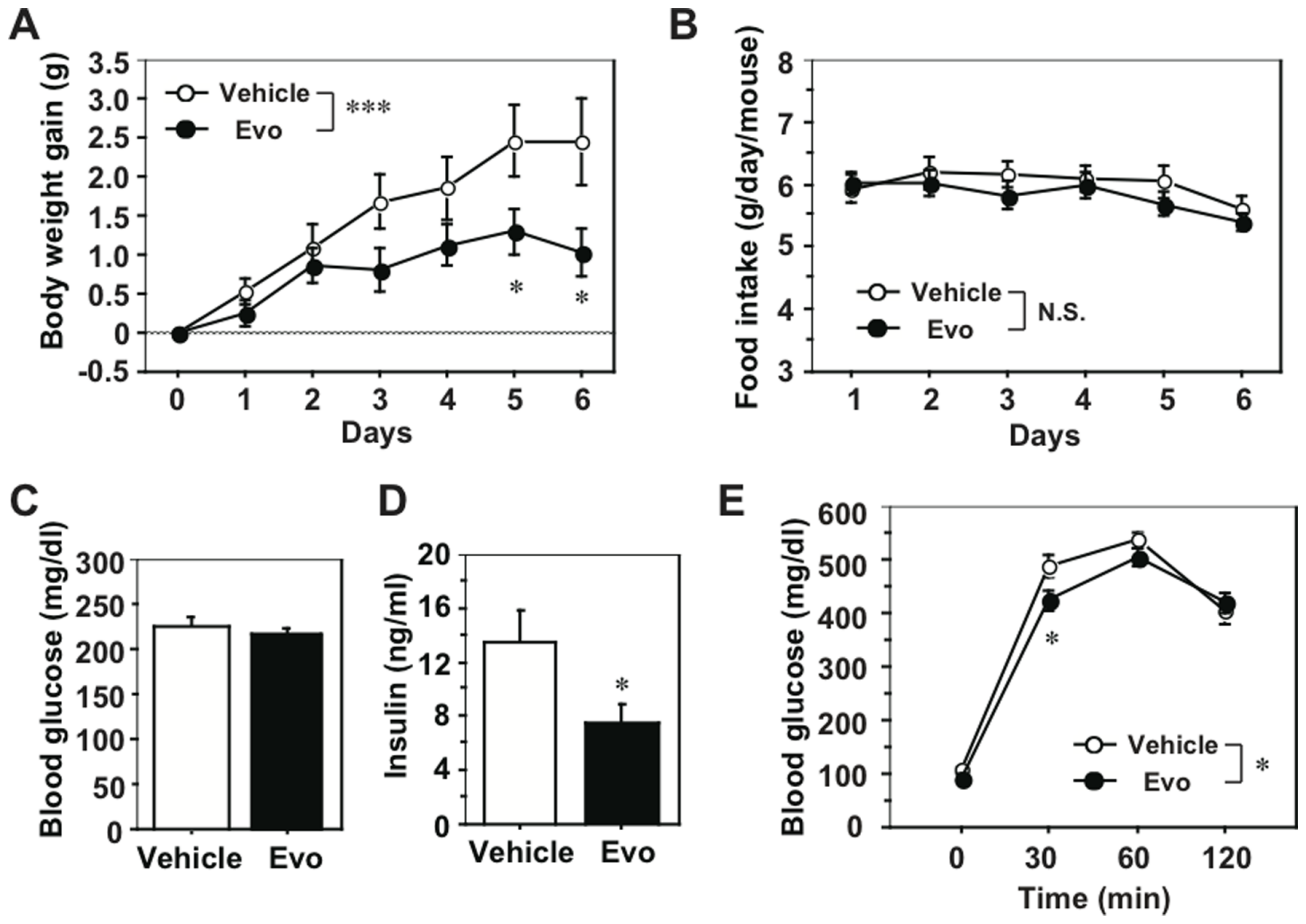
Effects of evodiamine on body weight, food intake, blood parameters and glucose tolerance in KK-Ay mice. Eight-weeks old female KK-Ay mice were injected i.p. daily with evodiamine (3 mg/kg body weight) or vehicle for 1 week. A, Body weight gain; B, food intake; C, fed glucose level; D, insulin level; E, IPGTT. After starvation for 17 h, mice were injected i.p. with glucose (1.5 mg/g body weight). Data are expressed as mean±SEM; *n* = 14 and 13 for body weight gain and food intake; *n* = 8 each for glucose and insulin levels; n = 9 and 8 for IPGTT, in vehicle and evodiamine groups, respectively. *p<0.05, ***p<0.001 *vs.* vehicle group.

**Figure 2:**
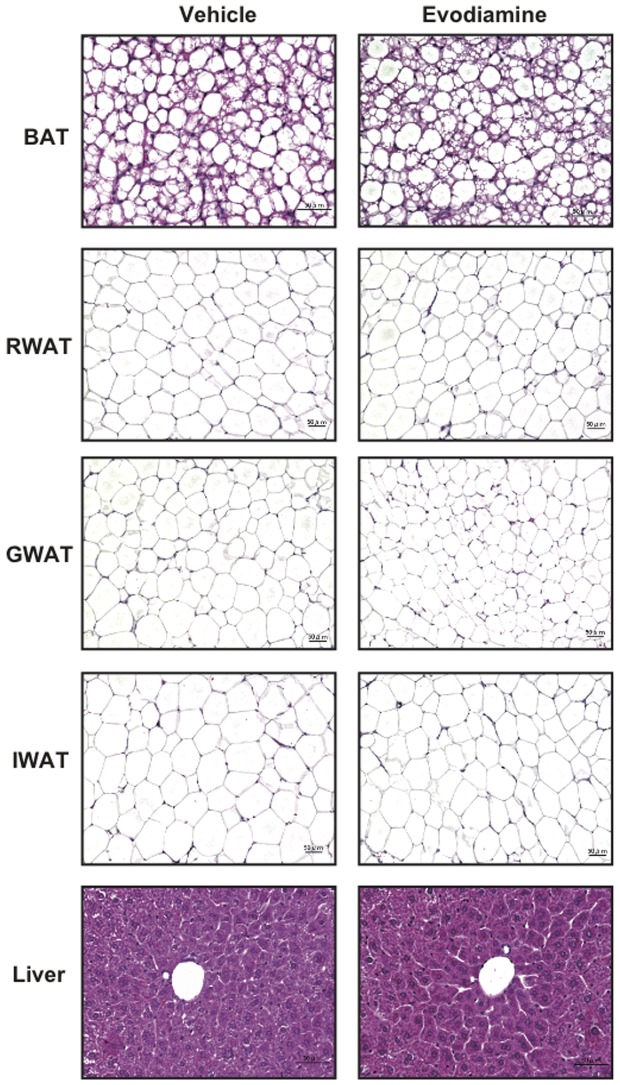
Histological analysis of BAT, RWAT, GWAT, IWAT and liver in KK-Ay mice treated with evodiamine. Tissue samples were collected from the mice treated with evodiamine for 7 days. Tissue sections of brown adipose tissue (BAT), retroperitoneal (RWAT), gonadal (GWAT), inguinal white adipose tissues (IWAT) and liver were stained with hematoxylin and eosin.

We examined the effects of evodiamine on the metabolic phenotypes associated with obese/diabetic states in KK-Ay mice. When mice were treated daily with evodiamine or vehicle (control) for one week, a significant decrease in body weight (BW) gain, but not in food intake, was found in the evodiamine group compared to the control group (Figs. 1A, B). As shown in Table 1, there was a significant reduction in tissue mass in several white fat depots, including inguinal white adipose tissue (IWAT) and retroperitoneal WAT (RWAT), in evodiamine-treated mice. There was no difference in liver weight between the two groups (Table 1). The same amount of glucose was fed to each group (Fig. 1C) but insulin levels were significantly lower in the evodiamine group compared to the control group (Fig. 1D), suggesting increased insulin sensitivity. Moreover, glucose intolerance (measured by an intraperitoneal (i.p.) glucose tolerance test) decreased in the evodiamine group compared to the control group (Fig. 1E). Histological analysis showed decreased trends of adipocyte size in WATs of the evodiamine group compared to the control group (RWAT, 3737±226 µm^2^ and 3503±303 µm^2^; gonadal WAT (GWAT), 1845±205 µm^2^ and 1403±152 µm^2^; IWAT, 4112±592 µm^2^ and 3844±308 µm^2^ in the control and evodiamine group, respectively; Fig. 2).

**Table 1:** Effect of evodiamine on tissue weight in KK-Ay mice.

	Vehicle (*n* = 5)	Evodiamine (*n* = 5)	*p*
Body weight (g)	38.0±1.2	37.3±0.5	0.6001
Tissue weight (mg)			
BAT	251±25	236±16	0.6305
IWAT	598±29	467±40	0.0300
GWAT	1584±145	1283±34	0.0786
RWAT	175±10	133±13	0.0372
MWAT	1017±122	911±23	0.4196
Kidney	174±7	179±6	0.5901
Liver	1901±54	1862±80	0.6979
Pancreas	330±21	362±17	0.2740
Heart	146±2	161±8	0.1163
Spleen	101±9	84±7	0.1722

Female 8-weeks old KK-Ay mice were injected i.p. daily with evodiamine (3 mg/kg body weight) or vehicle control. After 1 week, non-fasting samples of tissues and blood were collected. The left pads of inguinal (IWAT), gonadal (GWAT) and retroperitoneal (RWAT) white adipose tissues were used to measure tissue weight. BAT, brown adipose tissue; MWAT, mesenteric WAT.

### Evodiamine attenuates mTOR-S6K signaling and down-regulates IRS1 serine phosphorylation in WAT of obese/diabetic KK-Ay mice

**Figure 3:**
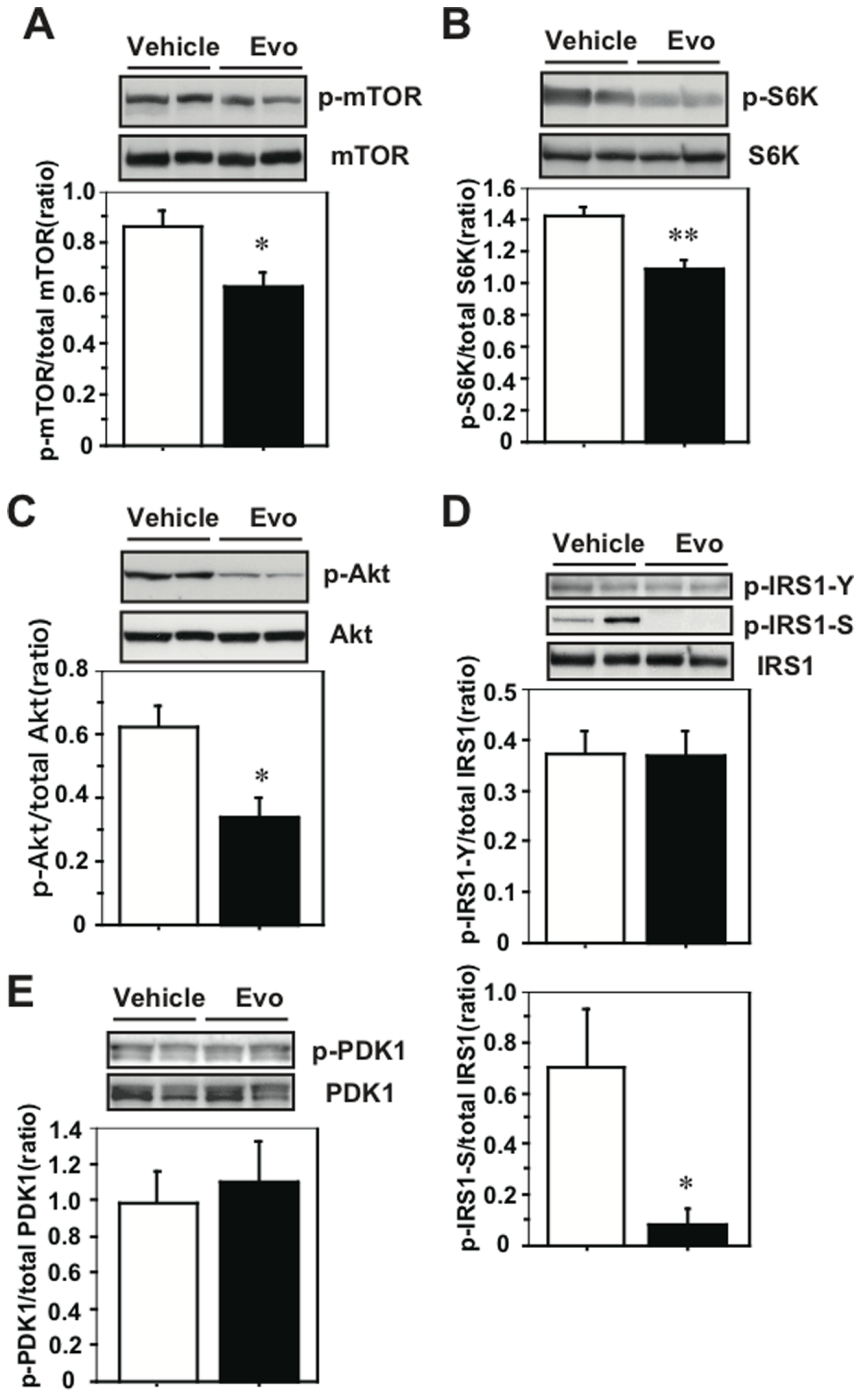
Effect of evodiamine on the mTOR-S6K signaling in the inguinal WAT (IWAT) of mice treated with evodiamine. Western blot analysis for mTOR (A), S6K (B), Akt (C), IRS1 (D) and PDK1 (E) were done with tissue lysates (30 µg protein) of IWAT from the KK-Ay mice treated with evodiamine for 7 days. Phosphorylation levels of mTOR Ser2448, S6K Thr389, Akt Ser473, IRS1 Tyr612 and Ser636/639, and PDK1 Ser241 were normalized to the total level of each protein. Data are expressed as mean±SEM (*n* = 5). *p<0.05, **p<0.01 *vs.* vehicle group.

**Figure 4:**
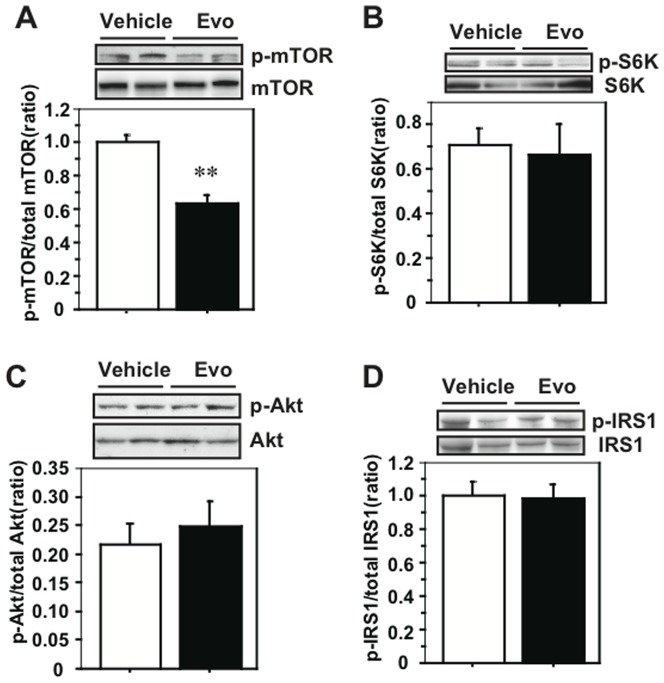
Effect of evodiamine on the mTOR-S6K signaling in the liver of mice treated with evodiamine. Western blot analysis for mTOR (A), S6K (B), Akt (C) and IRS1 (D) were done with tissue lysates (30–50 µg protein) of liver from the KK-Ay mice treated with evodiamine for 7 days. Phosphorylation levels of mTOR Ser2448, S6K Thr389, Akt Ser473 and IRS1 Ser636/639 were normalized to the total level of each protein. Data are expressed as mean±SEM (*n* = 5). **p<0.01 *vs.* vehicle group.

We examined the effects of evodiamine on the signaling of mTOR and IRS1 in the obese/diabetic mice because attenuated phosphorylation of Akt, an upstream kinase for mTOR, is present in the WAT of the evodiamine-treated mice, as reported [21]. There was no difference in mTOR Ser2448 phosphorylation in RWAT between the evodiamine and control groups (Fig. S1A in File S1) but there was a significant reduction of its level in IWAT of KK-Ay mice treated with evodiamine compared to the control group (Fig. 3A). There was a reduction in mTOR phosphorylation also in the WAT of UCP1-KO mice treated with evodiamine compared to the control group (Fig. S2A in File S1). There was no effect of evodiamine on mTOR phosphorylation or UCP1 protein level in the BAT of KK-Ay mice (Fig. S3 in File S1). Moreover, there were decreases in S6K Thr389 phosphorylation in IWAT and RWAT of evodiamine-treated mice (Fig. 3B and Fig. S1B in File S1). Similar to the results for the WAT of UCP1-KO mice treated with evodiamine (Figs. S2B, C in File S1), phosphorylation of Akt Ser473 and IRS1 Ser636/639 in the IWAT of KK-Ay mice was significantly lower (54% and 12% of the control, respectively) in the evodiamine group compared to the vehicle group (Figs. 3C, D) and phosphorylation of IRS1 Tyr612 and PDK1 Ser241 was not different between the two groups (Figs. 3D, E). There were significant decreases in the Akt and IRS1 serine phosphorylation in the RWAT of mice treated with evodiamine compared to the vehicle control (Figs. S1C, D in File S1). Although evodiamine administration reduces mTOR phosphorylation in liver, the phosphorylation levels of S6K, Akt and IRS1 were not different between the evodiamine and control groups (Fig. 4). Likewise, there were no differences in the phosphorylation levels of mTOR, S6K, Akt and IRS1 in gastrocnemius muscle (GM) between the evodiamine and control groups (Figs. S4A–D in File S1).

### Evodiamine inhibits insulin-stimulated phosphorylation of mTOR and S6K, reducing IRS1 serine phosphorylation in 3T3-L1 adipocytes

**Figure 5:**
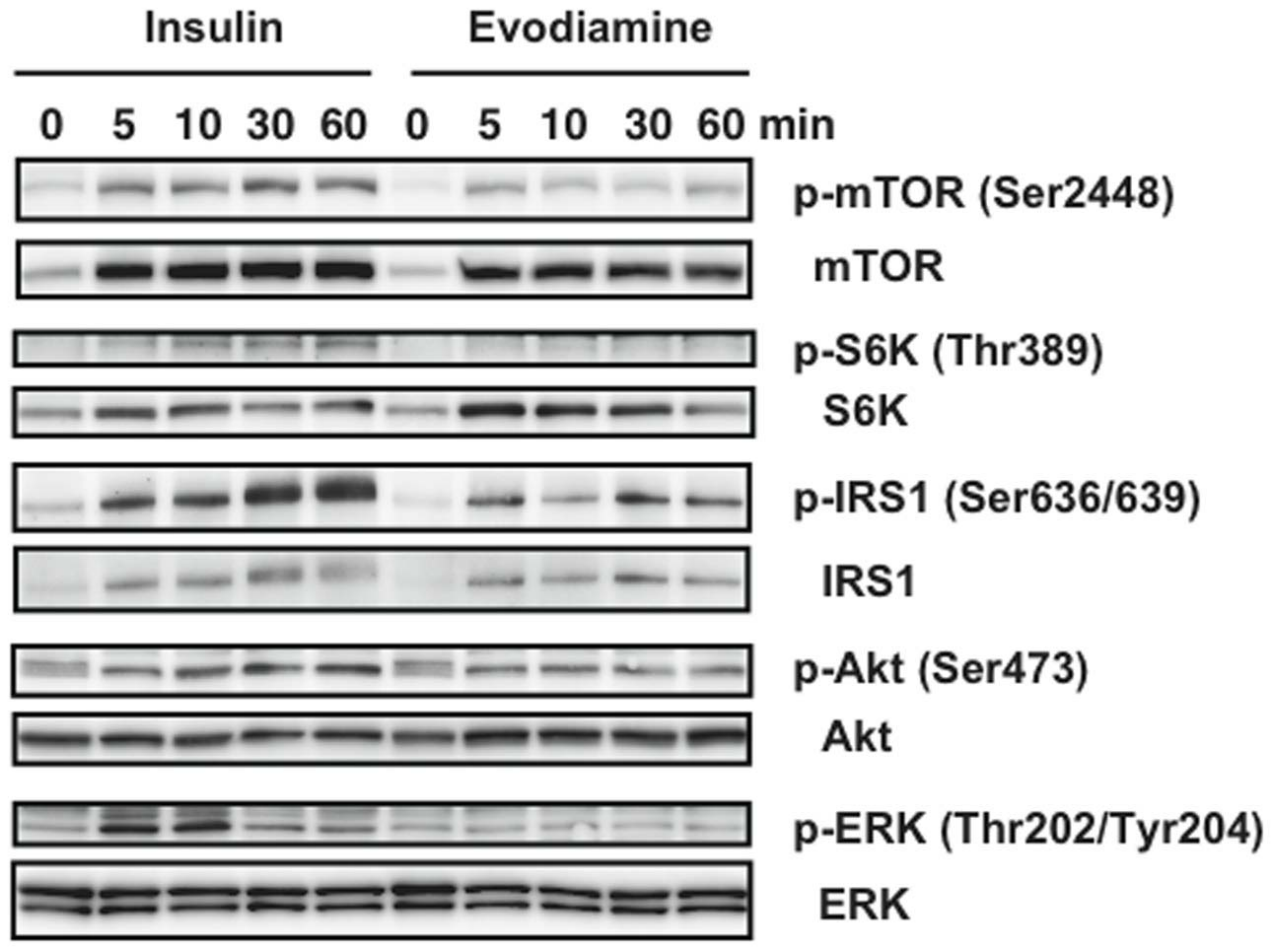
Effect of evodiamine and insulin on the mTOR-S6K signaling in differentiated adipocytes. After 3T3-L1 pre-adipocytes were differentiated into mature adipocytes, the cells were maintained in DMEM for 4 h and then stimulated by 20 nM insulin or 20 µM evodiamine for the indicated length of time. The cell lysates (15–30 µg protein) were analyzed for phosphorylation of mTOR Ser2448, S6K Thr389, IRS1 Ser636/639, Akt Ser473 and ERK Thr202/Tyr204 by Western blot analysis. Representative images of two independent experiments are shown.

**Figure 6:**
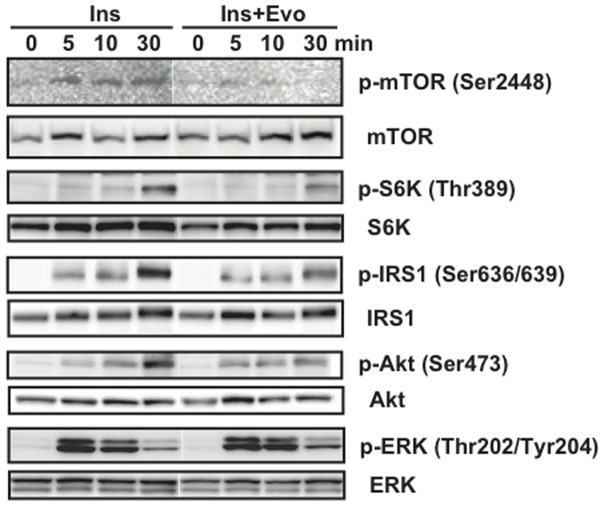
Effect of evodiamine on the insulin-stimulated activation of mTOR-S6K signaling in differentiated adipocytes. After 3T3-L1 pre-adipocytes were differentiated into mature adipocytes, the cells were maintained in DMEM for 4 h and then stimulated by 20 nM insulin with or without 20 µM evodiamine for the indicated length of time. The cell lysates (15–30 µg protein) were analyzed for phosphorylation of mTOR Ser2448, S6K Thr389, IRS1 Ser636/639, Akt Ser473 and ERK Thr202/Tyr204 by Western blot analysis. Representative images of two independent experiments are shown.

**Figure 7:**
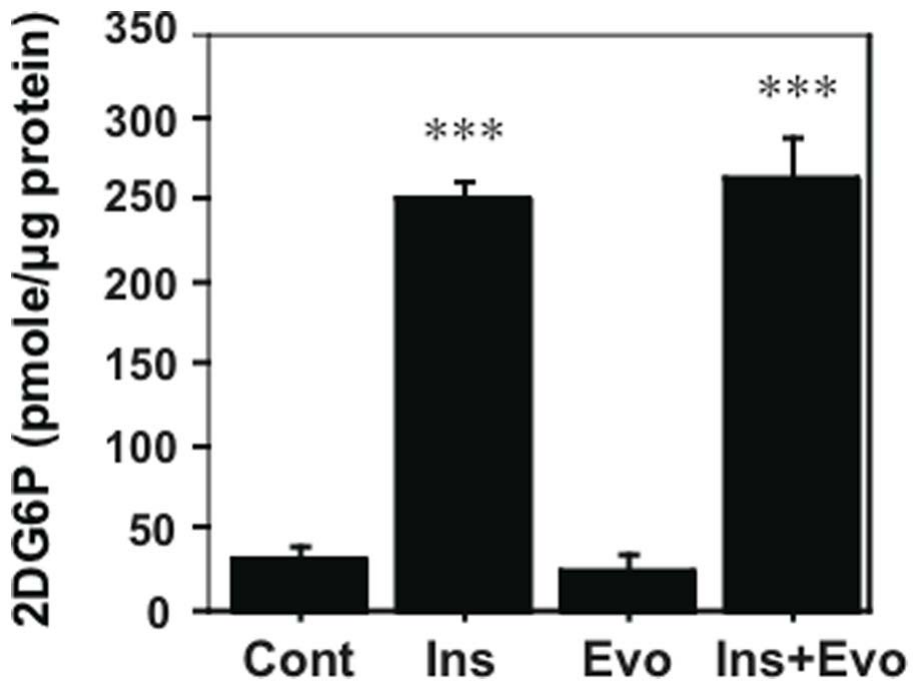
Effect of evodiamine and insulin on glucose uptake in differentiated adipocytes. After 3T3-L1 pre-adipocytes were differentiated into mature adipocytes, the cells were maintained in DMEM for 6 h and then stimulated by 20 nM insulin or 20 µM evodiamine alone, or in combination with insulin and evodiamine for 40 min and with 1 µM 2DG for the last 20 min. The 2DG6P content in each cell sample was measured by using glucose uptake colorimetric assay kit. Data are expressed as mean±SEM (*n* = 3–4). ***p<0.001 *vs.* control group without stimulation.

**Figure 8:**
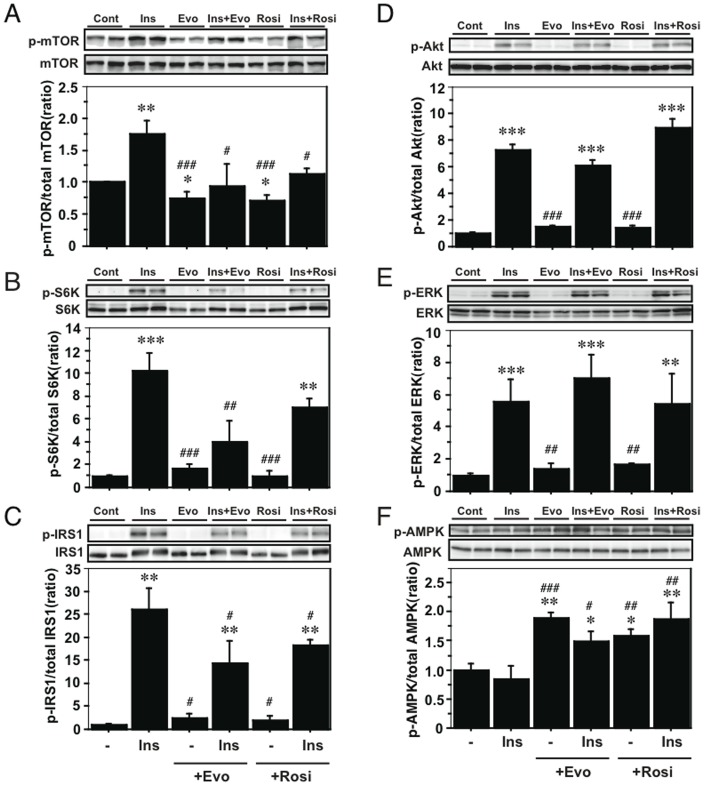
Effect of evodiamine and rosiglitazone on the mTOR-S6K signaling in adipocytes with or without insulin stimulation. After 3T3-L1 pre-adipocytes were differentiated into mature adipocytes, the cells were maintained in DMEM for 4 h and then stimulated by 20 nM insulin, 20 µM evodiamine or 20 µM rosiglitazone alone, or in combination with insulin and evodiamine or rosiglitazone for 30 min. The cell lysates (15–30 µg protein) were analyzed for phosphorylation of mTOR Ser2448, S6K Thr389, IRS1 Ser636/639, Akt Ser473, ERK Thr202/Tyr204 and AMPK Thr172 by Western blot analysis. Phosphorylation levels were normalized to the total level of each protein. Data are expressed as mean±SEM (*n* = 3–5). *p<0.05, **p<0.01, ***p<0.001 *vs.* control group without stimulation. ^#^ *p*<0.05, ^##^ *p*<0.01, ^###^ *p*<0.001 *vs.* insulin stimulation.

To assess the direct effects of evodiamine on adipocytes, we examined the effects of insulin and evodiamine on stimulation of the mTOR-S6K signaling pathway using differentiated 3T3-L1 adipocytes. As well as the stimulation of IRS1 and Akt phosphorylation, insulin-stimulated phosphorylation of mTOR and S6K was time-dependent; however, evodiamine had little effect on their phosphorylation (Fig. 5). Insulin, but not evodiamine, stimulated ERK phosphorylation transiently in mature adipocytes. This was in contrast to the effect of evodiamine in 3T3-L1 pre-adipocytes, in which this compound markedly increased ERK phosphorylation (Fig. S5 in File S1). In the presence of insulin stimulation, evodiamine seemed to suppress the phosphorylation of mTOR, S6K, IRS1 and Akt, except for ERK phosphorylation in adipocytes (Fig. 6), although evodiamine did not affect glucose uptake in adipocytes (Fig. 7). Insulin-induced serine phosphorylation of Akt and IRS1 was suppressed by evodiamine in pre-adipocytes (Fig. S5 in File S1). We evaluated the effects of insulin and evodiamine on phosphorylation of these molecules in adipocytes. As shown in Figures 8A–E, insulin markedly increased phosphorylation of mTOR (1.8-fold), S6K (10.3-fold), IRS1 (26.1-fold), Akt (7.3-fold) and ERK (5.6-fold) in 3T3-L1 adipocytes compared to the control, whereas evodiamine had no significant effect on phosphorylation of these signaling molecules, except for an inhibitory effect on mTOR phosphorylation. By contrast, evodiamine suppressed insulin-stimulated phosphorylation of mTOR, S6K and IRS1 in adipocytes significantly (Figs. 8A–C). Evodiamine did not significantly affect the levels of insulin-stimulated Akt or ERK phosphorylation in adipocytes (Figs. 8D, E), although their phosphorylation was affected considerably by evodiamine in pre-adipocytes; i.e. Akt phosphorylation was inhibited but ERK phosphorylation was enhanced (Fig. S5 in File S1) [21].

We examined the effects of rosiglitazone, an anti-diabetic compound, on the phosphorylation of these signaling molecules compared to the effects of insulin and evodiamine in adipocytes. Similar to evodiamine, rosiglitazone alone did not affect phosphorylation of S6K, IRS1, Akt and ERK (except mTOR) in the cells but, like evodiamine, rosiglitazone suppressed insulin-stimulated phosphorylation of mTOR and IRS1 in adipocytes significantly (Figs. 8A, C), although its effect on S6K phosphorylation in insulin-stimulated cells did not reach significance (Fig. 8B). There was no significant effect of rosiglitazone on insulin-stimulated Akt or ERK phosphorylation in adipocytes (Figs. 8D, E).

### Evodiamine stimulates phosphorylation of AMP-activated protein kinase (AMPK) in 3T3-L1 adipocytes and WAT

**Figure 9:**
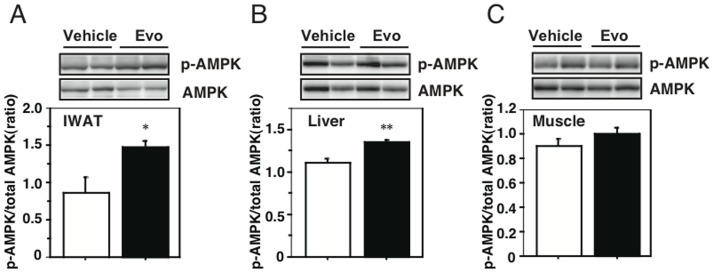
Effect of evodiamine on AMPK phosphorylation in the WAT, liver and sleletal muscle of mice treated with evodiamine. Western blot analysis for AMPK were done with tissue lysates (30 µg protein) of IWAT (A), liver (B) and gastrocnemius muscle (C) from the KK-Ay mice treated with evodiamine for 7 days. Phosphorylation levels of AMPK Thr172 were normalized to its total level. Data are expressed as mean±SEM (*n* = 5). *p<0.05, **p<0.01 *vs.* vehicle group.

From the results in Figures 8D–E, Akt and ERK may not be the upstream effectors for mTOR-S6K signaling in mature adipocytes stimulated by evodiamine. So, we finally examined an involvement of AMPK in the mechanism by which evodiamine affects mTOR signaling pathway, because its phosphorylation is negatively regulated by the activity of AMPK, which regulates various cellular processes including glucose and lipid metabolism [22], [23]. As well as rosiglitazone, evodiamine significantly stimulated AMPK Thr172 phosphorylation (1.9-fold) in 3T3-L1 adipocytes compared to the control, whereas insulin had no effect on its phosphorylation (Fig. 8F). Significant increaes in AMPK phosphorylation were also detected in the WAT and liver, but not in gastrocnemius muscle (GM), of KK-Ay mice treated with evodiamine compared to the vehicle control (Figs. 9A–C), supporting the decreases of mTOR phosphorylation in these tissues (Figs. 3A and 4A).

## Discussion

Evodiamine is a multipotent compound that has a wide variety of bioactivities, such as anti-obesity and anti-tumor effects [17], [19]. Earlier, we reported that a dietary supplementation of evodiamine inhibited adipocyte differentiation and several obesity-associated phenotypes, such as insulin resistance in mice lacking UCP1 thermogenesis, indicating that UCP1-independent roles of this compound improve health under conditions of excess caloric intake [21]. Although indirect calorimetric analysis did not detect the effect of evodiamine on energy expenditure, a precise analysis of respiratory quotient (RQ) values indicated that a higher frequency of higher RQ values was present in evodiamine-injected mice compared to vehicle-injected animals. This indicates that evodiamine affects glucose homeostasis in the whole body (Fig. S6 in File S1). We also detected the effect of evodiamine on glucose tolerance and insulin sensitivity in obese/diabetic KK-Ay mice, in which decreases in BW gain and WAT mass were observed in the evodiamine-treated KK-Ay mice compared to the vehicle control. These results were consistent with the Bak's study that evodiamine prevented BW gain and insulin resistance in db/db mice [24]. Reduced fat accumulation in several depots of WAT might contribute to the improvement of insulin sensitivity in the obese/diabetic mice treated with evodiamine, because hypertrophy of WATs is known to cause the decrease in insulin sensitivity in animals.

The recent advance in understanding the role of the mTOR-S6K signaling pathway is of great interest in relation to the effects of evodiamine, because this signaling pathway is activated in response to growth factors, insulin or nutrients such as glucose and amino acids [12], [14] and its increased activity leads to serine phosphorylation of IRS1, creating a negative feedback loop to insulin signaling that attenuates insulin sensitivity [14], [15]. As expected, there is a significant decrease in phosphorylation of S6K in the WATs of evodiamine-treated KK-Ay mice. This down-regulation of S6K activity appears to lead to reduced serine phosphorylation of IRS1 in tissues, contributing to the improvement of insulin sensitivity in KK-Ay mice treated with evodiamine. Akt is also a key molecule for the intracellular signal cascade in the regulation of many cellular activities, including growth, glucose metabolism and adipogenesis [25], [26]. Akt activity is regulated by various pathways and insulin signaling via IRS1 and PDK1 [11], [12]. IRS1 protein contains multiple sites for tyrosine phosphorylation and both Tyr612 and Tyr632 are important for IRS1 to fully activate PI3K and mediate translocation of GLUT4 in response to insulin in adipocytes [27]. PDK1 may also stimulate GLUT4 translocation via atypical protein kinase C (PKC) activation without Akt activation, because PDK1 phosphorylates the PKC isoforms in a PI3K-dependent manner [28] and glucose uptake can be regulated through the Akt-independent PI3K-PKC pathway in adipocytes [29], [30]. In the present study, there was a significant decrease in Akt phosphorylation in WATs of evodiamine-treated KK-Ay mice compared to the vehicle control, which is similar to the result in mice fed an evodiamine diet [21]. Nevertheless, glucose metabolism was improved in these evodiamine-treated mice. Moreover, evodiamine did not significantly affect Akt phosphorylation or glucose uptake in adipocytes *in vitro*. So, the contribution of decreased Akt phosphorylation in WATs to the regulation of glucose uptake may be little in the evodiamine-treated mice. Because phosphorylation of IRS1 Tyr612 and PDK1 Ser241 in the WAT was not changed by treatment with evodiamine (Fig. 3), the decrease in Akt phosphorylation could have been induced by signaling pathways other than the main pathway of insulin signaling.

mTOR is the catalytic subunit of complexes mTORC1 and mTORC2. Akt stimulates mTORC1 activity but, at the same time, is a target of mTORC2 signaling [12]. So, the decrease in Akt phosphorylation could be mediated by reduction of mTORC2 activity. The meaning of this down-regulation of Akt phosphorylation in WATs is not clear, but its reduction might have contributed to suppression of adipocyte differentiation, because adipogenesis is blocked in cultured cells or mice lacking Akt [26]. The insulin-stimlated Akt phosphorylation is attenuated by evodiamine in pre-adipocytes but not in mature adipocytes. Because the basal phosphorylation level of Akt is much higher in pre-adipocytes than in mature adipocytes, it is possible that the decreased level of Akt phosphorylation in WATs of mice treated with evodiamine was owing to its reduction in pre-adipocytes, rather than in mature adipocytes, existing in the depots. It is also possible that reduction of mTORC2 activity might contribute to the improvement of glucose tolerance in the evodiamine-treated KK-Ay mice, as glucose uptake into WAT is increased in mice with whole-body deletion of Rictor, the mTORC2 subunit, whereas disruption of mTORC2 in liver causes glucose intolerance [31]. Chronic treatment (2–4 weeks) with rapamycin, the mTOR inhibitor, causes glucose intolerance and insulin resistance in mice [31], [32] but evodiamine appears not to be included, because administration of this compound for 2 months improved glucose tolerance and insulin resistance associated with diet-induced obesity in mice [21], in which phosphorylation levels of mTOR and IRS1 were decreased in evodiamine-treated WAT (Fig. S2 in File S1). Likewise, mTORC1 activity appears to be attenuated in the WATs of KK-Ay mice treated with evodiamine, because the phosphorylation of S6K, a target of mTORC1 signaling, is reduced considerably in adipose tissues, which blocks serine phosphorylation of IRS1. Similar effects of evodiamine on mTOR-S6K signaling were confirmed in adipocytes *in vitro*. In contrast to the insulin effects that stimulated serine phosphorylation of IRS1 and Akt as well as activation of mTOR-S6K signaling, evodiamine alone does not affect these molecules except for the inhibitory effect on mTOR phosphorylation in adipocytes. However, evodiamine inhibited insulin-stimulated phosphorylation of mTOR and S6K, leading to suppression of IRS1 serine phosphorylation in adipocytes, suggesting evodiamine acts to improve insulin resistance by repressing activation of mTOR-S6K signaling by insulin.

AMPK is a major cellular energy sensor controlling glucose and lipid metabolism [22], [23]. AMPK is activated in response to a variety of stimuli, including metabolic stress that generates an increase in the AMP/ATP ratio. Genetic and pharmacologic studies demonstrated that AMPK is required for maintaining glucose homeostasis [33]. Therefore, AMPK is now considered to be a potential pharmacologic target for improving insulin resistance, diabetes and metabolic syndrome. Ching et al. have recently reported that evodiamine increased AMPK phosphorylation in endothelial cells [34]. In the present study, we found that evodiame significantly stimulated AMPK phosphorylation in mature adipocyte culture and in WAT of KK-Ay mice. The increased phosphorylation of AMPK was also detected in liver in a lesser extent, but not in skeletal muscle, in the evodiamine-treated KK-Ay mice compared to the vehicle control. AMPK down-regultes mTOR signaling pathway via phosphorylation of tuberous sclerosis complex 2, which inhibits cell growth [12], [35]. Therefore, it is plausible that evodiamine inhibits mTOR-S6K signaling through AMPK phosphorylation in adipocytes. Because increased AMPK phosphorylation and decreased mTOR phosphorylation were also detected in liver, but not in skeletal muscle, of the evodiamine-treated mice, evodiamine might affect glucose metabolism and insulin sensitivity in various tissues including WAT and liver.

The anti-diabetic compound rosiglitazone has effects similar to those of evodiamine on AMPK phosphorylation and insulin-stimulated phosphorylation of mTOR and IRS1 in adipocytes, although the effects of these two compounds on adipogenesis are opposite; i.e. evodiamine inhibits but rosiglitazone stimulates the cellular process [21], [22]. It is noteworthy that the difference between adipocytes and pre-adipocytes in responsiveness to evodiamine, as indicated from the results of ERK and Akt phosphorylation, suggests a change in the mode of evodiamine action corresponding to the differentiation of pre-adipocytes into mature adipocytes. So, evodiamine might exhibit its improved effects against obesity and insulin resistance in different modes of action in pre-adipocytes and adipocytes.

Our data suggest evodiamine improves glucose tolerance and prevents progress of insulin resistance associated with obese/diabetic states, at least in part, through AMPK activation followed by inhibition of mTOR-S6K signaling and IRS1 serine phosphorylation in adipocytes. The anti-obesity effect of evodiamine may also contribute indirectly to prevention against insulin resistance. Considering that inhibition of mTOR-S6K signaling has attracted a great deal of attention in connection with health and longevity [12], [36], [37], evodiamine might be a unique compound able to combat obesity and age-related diseases; however, the effects of evodiamine on energy metabolism in other metabolic tissues remain to be clarified.

## Methods

### Ethics statement

This study was performed in strict accordance with the recommendations in Fundamental Guidelines for Proper Conduct of Animal Experiment and Related Activities in Academic Research Institutions under the jurisdiction of the Ministry of Education, Culture, Sports, Science and Technology, Japan. The protocol was approved by the Institutional Animal Care and Use Committee in Chubu University (approval number: 2310039 and 2410003).

### Experimental animals

KK-Ay mice were obtained from CLEA Japan, Inc. and maintained at 23±1°C under artificial lighting for 12 h/day and provided a standard chow (11.6% kcal from fat; Diet no. CE-2, CLEA Japan, Inc.) and tap water *ad libitum*. Eight-weeks old female mice were administered evodiamine by i.p. injection (3 mg/kg BW, KISIDA CHEMICAL CO., LTD, Japan) or vehicle (10% (v/v) DMSO, 10% (v/v) Tween80, 80% (w/v) NaCl) once a day for 7 days. Compound solutions were kept at ∼35°C and injected i.p. at a dose of 100 µl/g BW (3 mg/ml). BW and food intake were recorded each day. After 1 week, ten mice for tissue analyses were euthanized by cervical dislocation; their blood and tissue samples were recovered and stored at −80°C. Total seventeen mice in two independent experiments for glucose tolerance test were fasted for 17 h after the final injection of evodiamine or vehicle.

### Biochemical analysis

Blood samples were collected from a tail vein and used immediately to determine the glucose level with a glucometer (OneTouch Ultra, Johnson & Johnson K.K., Tokyo, Japan). Insulin levels were measured with serum and commercial assay kits (Ultrasensitive insulin ELISA, Mercodia, Winston Salem NC, USA). An i.p. glucose tolerance test (IPGTT) using 1.5 mg glucose/g BW was done after starvation for 17 h. The blood glucose level was measured before the injection of glucose at time zero and at 30, 60 and 120 min later.

### Cell culture

3T3-L1 cells (ATCC, CL173, DS Pharma Biomedical Co., Osaka, Japan) were grown in Dulbecco's modified Eagle medium (DMEM; Wako Pure Chemical, Osaka, Japan) containing 10% (v/v) calf serum (BioWest). Adipocyte differentiation was done essentially as described but with minor modifications [38]. Briefly, 2 days post confluence, the medium was changed to DMEM containing 10% (v/v) fetal bovine serum (ICN Biomedicals, Aurora, OH, USA), 10 µg/ml insulin, 1 µM dexamethasone, 0.5 mM 3-isobutyl-1-methylxanthine and 10 µM pioglitazone. Dexamethasone, 3-isobutyl-1-methylxanthine and pioglitazone were withdrawn after 2 days of exposure and insulin was withdrawn after 4 days. On day 6, most of the pre-adipocytes were differentiated into adipocytes. The cells were maintained in DMEM for 4 h and then stimulated by 20 nM insulin, 20 µM evodiamine or 20 µM rosiglitazone (Wako).

### Protein analysis

Western blot analysis was done as described with the total tissue lysates or whole-cell lysates recovered from the WAT or 3T3-L1 adipocytes [39]. The concentration of protein in the lysates was measured with the BCA protein assay (Pierce Biotechnology, Rockford, IL, USA). Equal amounts of protein (15–50 µg) were separated by electrophoresis in SDS/4–20% (w/v) polyacrylamide gels (Daiichi Pure Chemicals, Tokyo, Japan) and transferred electrophoretically onto Immobilon polyvinylidene difluoride membranes (Millipore, Bedford, MA, USA). The membranes were incubated with specific antibodies against Akt, phospho-Ser473 Akt, mTOR, phospho-Ser2448 mTOR, IRS1, phospho-Ser636/639 IRS1, p70S6 kinase, phospho-Thr389 p70S6 kinase, PDK1, phospho-Ser241 PDK1, Erk1/2, phospho-Thr202/Tyr204 Erk1/2, AMPK, phospho-Thr172 AMPK (Cell Signaling Technology, Danvers, MA, USA) or phospho-Tyr612 IRS1/2 (Santa Cruz). After the secondary antibody reaction at 4°C overnight, specific signals were detected using Immobillon Western Detection Reagents (Merck Japan, Tokyo). The resulting images were quantified with NIH Image (version 1.63) software.

### Glucose uptake analysis

Glucose uptake into 3T3-L1 adipocytes was evaluated according to the method of Saito et al. [40] with minor modification. After the adipocytes were maintained in DMEM for 6 h, cells were washed with Krebs Ringer Phosphate Hepes (KRPH) buffer (20 mM HEPES, 5 mM KH2PO4, 1 mM MgSO4, 1 mM CaCl2, 136 mM NaCl, 4.7 mM KCl, pH 7.4) containing 2% bovine alubumin (fatty acid-free, NACALAI TESQUE, INC. KYOTO, JAPAN) three times and incubated in the KRPH buffer for 40 min. The cells were then stimulated by 20 nM insulin with or without 20 µM evodiamine for 40 min and with 1 µM 2-Deoxy-D-glucose (2DG, Sigma-Aldrich, St. Louis, MO, USA) for the last 20 min. Cells were washed with ice-cold phosphate buffered saline containing 5 µM cytochalasin B (Sigma-Aldrich) three times and collected in 1 ml of 10 mM Tris-HCl (pH 8.0). After sonication, the homogenates were heated at 80°C for 15 min and centrifuged at 15,000 g for 20 min at 4°C. A portion of the resulting supernatant was diluted 10 times with 10 mM Tris-HCl (pH 8.0) and analyzed for 2DG6P content by using glucose uptake colorimetric assay kit (BioVision, Milpitas, CA, USA). The 2DG6P content in each sample was corrected by subtracting the value derived from the untreated cells without 2DG addition.

### Histological analysis

Immediately after removal, tissue pieces were fixed by immersion at 4°C in 10% (v/v) formaldehyde in neutral buffer solution (Kishida Chemical), dehydrated, cleared and embedded in paraffin. Paraffin sections (4-µm thick) were stained with hematoxylin and eosin. All observations were done with a fluorescence microscope (Biorevo BZ-9000; Keyence, Tokyo, Japan). The average cell size of adipocytes in WAT was calculated by dividing the chosen microscopic area by the total cell number in the area.

### Statistical analysis

Data were expressed as mean±SEM. Differences between groups were assessed by analysis of variance (ANOVA) or repeated measure ANOVA. The level of statistically significant difference was set at *p*≤0.05.

## Supporting Information

File S1 Methods S1, Animal experiments using UCP1-knockout (KO) mice and cell culture experiments using 3T3-L1 pre-adipocytes. References S1, References for supplementary methods. Figure S1, Effect of evodiamine on mTOR-S6K signaling in the RWAT of KK-Ay mice treated with evodiamine. Western blot analysis of mTOR (A), S6K (B), Akt (C) and IRS1 (D) was done with tissue lysates (30 µg protein) of retroperitoneal WAT from KK-Ay mice treated with evodiamine for 7 days. Phosphorylation levels of mTOR Ser2448, S6K Thr389, Akt Ser473 and IRS1 Ser636/639 were normalized to the total level of each protein. Data are expressed as mean±SEM (*n* = 5). *p<0.05 *vs.* vehicle group. Figure S2, Effect of evodiamine on phosphorylation of mTOR, Akt and IRS1 in the WAT of obese UCP1-KO mice. Western blot analysis for mTOR (A), Akt (B) and IRS1 (C) was done with tissue lysates (50 µg protein) of epididymal WAT from the UCP1-KO mice fed a high-fat diet with or without evodiamine for 2 months as described in Supplemental Methods. Phosphorylation levels of mTOR Ser2448, Akt Ser473 and IRS1 Ser636/639 were normalized to the total level of each protein. Data are expressed as mean±SEM (*n* = 4). *p<0.05 *vs.* control group. Figure S3, Effect of evodiamine on phosphorylation of mTOR and UCP1 in the BAT of KK-Ay mice treated with evodiamine. Western blot analysis for mTOR (A) and UCP1 (B) was done with tissue lysates (30 µg protein) of BAT from KK-Ay mice treated with evodiamine for 7 days. Levels of mTOR Ser2448 phosphorylation and UCP1 protein were normalized to the total mTOR and tubulin levels, respectively. Data are expressed as mean±SEM (*n* = 5). Figure S4, Effect of evodiamine on phosphorylation of mTOR, S6K, Akt and IRS1 in the gastrocnemius muscle (GM) of KK-Ay mice treated with evodiamine. Western blot analysis for mTOR (A), S6K (B), Akt (C) and IRS1 (D) was done with tissue lysates (30 µg protein) of GM from KK-Ay mice treated with evodiamine for 7 days. Phosphorylation levels of mTOR Ser2448, S6K Thr389, Akt Ser473 and IRS1 Ser636/639 were normalized to the total level of each protein. Data are expressed as mean±SEM (*n* = 5). Figure S5, Effect of evodiamine on phosphorylation of ERK, Akt and IRS1 in pre-adipocytes. 3T3-L1 pre-adipocytes were serum-deprived for 4 h and then treated with 20 µM evodiamine for 1 h and with 20 nM insulin for the last 10 min. Western blot analysis for ERK, Akt and IRS1 were done with cell lysates (30 µg protein). Figure S6, Measurement of oxygen consumption (VO_2_) and respiratory quotient (RQ) in UCP1-KO mice with or without treatment with evodiamine. VO_2_ (A) and RQ (B) were measured for 24 h in male mice injected with vehicle or evodiamine (3 mg/kg BW) at 14:00 h. C, Data on RQs were analyzed as relative cumulative frequency (PRCF). Each curve represents 1365 measurements of RQ from three mice for each group as described in Supplemental Methods. Data are expressed as mean±SEM (*n* = 3 for each group). ***p<0.001 *vs.* vehicle group.
(DOCX)
