# Supplementary material for: Evodiamine Inhibits Insulin-Stimulated mTOR-S6K Activation and IRS1 Serine Phosphorylation in Adipocytes and Improves Glucose Tolerance in Obese/Diabetic Mice
Source: PLoS One. 2013 Dec 31;8(12):e83264. doi: 10.1371/journal.pone.0083264 (PMC3877036; doi:10.1371/journal.pone.0083264)

**Supporting Information**

**Supplementary Methods**

*Experimental animals.* Congenic UCP1-knockout (KO) mice on a C57BL/6J background were prepared as previously described [1]. The mice were maintained under artificial lighting for 12 h per day and provided a standard chow (11.6% kcal from fat; Diet No. CE-2, CLEA Japan, Inc.) and tap water *ad libitum* in our animal facility at 23±1°C. The experiment of evodiamine diet using UCP1-KO mice was performed as previously described [2]. Briefly, the 4-month-old mice were fed a high-fat diet (HF: 41.9% kcal from fat, Diet No. B15040, CLEA Japan, Inc) with or without 300 mg evodiamine per kg food (Kishida Chemical, Osaka, Japan) for 2 months. The 6-month-old mice were sampled and the tissues were stored at -80°C until use. All experiments were performed in accordance with our institutional guidelines for the care and use of research animals.

*Indirect calorimetry.* Whole body oxygen consumption (VO_2_) and respiratory quotient (RQ) were measured by using an O_2_ analyzer (MM-102R; Muromachi Kikai, Ltd., Tokyo, Japan). Adult male UCP1-KO mouse was housed in an airflow chamber at a room temperature of 24°C with standard chow and tap water *ad libitum* for 72 h, and the air samples were taken every 3 minutes for analysis except once per hour for calibration (228 data points per 12 h in each light and dark phase). After the basal oxygen consumption in each mouse was determined during the first 24 h, the measurement was continued to obtain the data on vehicle and evodiamine treatment during the second and last 24 h, respectively. The mice were intraperitoneally administered vehicle (10% Tween80, 10% DMSO and 80% saline) or evodiamine (3 mg/kg BW, Kishida Chemical, Osaka, Japan) at 14:00. Data on RQs was also analyzed as percent relative cumulative frequency (PRCF) to address the slight differences in the number of data points collected between mouse groups and to facilitate comparison of data sets from experiment to experiment as previously described [3]. The resulting curves, which were derived from 1365 measurements from an experimental group of 3 mice, provided a sensitive indicator of slight shifts to lower or higher RQ.

*Cell culture.* 3T3-L1 pre-adipocytes, which are mouse embryonic fibroblasts capable of differentiating into adipocytes, were grown in Dulbecco’s modified Eagle’s medium (DMEM; Invitrogen, Grand Island, NY, USA) containing 10% calf serum (CS; ICN Biomedicals, Aurora, OH, USA). Two days postconfluence, the pre-adipocytes were washed with serum-free DMEM once and cultured in the fresh DMEM for 4 h. The cells were then treated with 20μM evodiamine for 1 h and with 20nM insulin for the last 10 min.

*Protein analysis.* Western blot analyses were carried out using the total tissue lysates or whole cell lysates recovered from adipose tissues or 3T3-L1 pre-adipocytes, as described previously [4]. The concentrations of protein in the lysates were measured by using a BCA protein assay (Pierce Biotechnology, Rockford, IL). Equal amounts of protein (15~50 µg) were separated on 4–20% SDS-polyacrylamide gels (Daiichi Pure Chemicals, Tokyo, Japan) and transferred onto Immobilon polyvinylidene difluoride membranes (Millipore, Bedford, MA, USA). The membranes were incubated with specific antibodies against Akt, phospho-Ser473 Akt, mTOR, phospho-Ser2448 mTOR, IRS1, phospho-Ser307 IRS1, phospho-Ser636/639 IRS1, p70S6 kinase, phospho-Thr389 p70S6 kinase, Erk1/2, phospho-Thr202/Tyr204 Erk1/2, α/β-tubulin (Cell Signaling Technology, Danvers, MA, USA) or UCP1 (Abnova, Taipei City, Taiwan). After performing the secondary antibody reaction at 4°C overnight, specific signals were detected using Immobillon Western Detection Reagents (Merck Japan, Tokyo). The resulting images were quantified with NIH Image (version 1.63).

# **Supplementary References**

1. Enerback S, Jacobsson A, Simpson EM, Guerra C, Yamashita H, Harper ME, Kozak LP (1997) Mice lacking mitochondrial uncoupling protein are cold-sensitive but not obese. Nature 387, 90-94.

2. Wang T, Wang Y, Kontani Y, Kobayashi Y, Sato Y, Mori N, Yamashita H (2008) Evodiamine improves diet-induced obesity in a uncoupling protein-1-independent manner: involvement of antiadipogenic mechanism and extracellularly regulated kinase/mitogen-activated protein kiase signaling. Endocrinol. 149, 358-366.

3. Liu X, Rossmeisl M, McClaine J, Riachi M, Harper ME, Kozak LP (2003) Paradoxical resistance to diet-induced obesity in UCP1-deficient mice. J. Clin. Invest. 111, 399-407.

4. Summers SA, Lipfert L, Birnbaum MJ (1998) Polyoma middle T antigen activates the Ser/Thr kinase Akt in a PI3-kinase-dependent manner. Biochem. Biophys. Res. Commun. 246, 76-81.

**Supplementary Figure Legends**

Figure S1.

Effect of evodiamine on mTOR-S6K signaling in the RWAT of KK-Ay mice treated with evodiamine. Western blot analysis of mTOR (A), S6K (B), Akt (C) and IRS1 (D) was done with tissue lysates (30 µg protein) of retroperitoneal WAT from KK-Ay mice treated with evodiamine for 7 days. Phosphorylation levels of mTOR Ser2448, S6K Thr389, Akt Ser473 and IRS1 Ser636/639 were normalized to the total level of each protein. Data are expressed as mean±SEM (*n* = 5). **p* <0.05 *vs*. vehicle group.

Figure S2.

Effect of evodiamine on phosphorylation of mTOR, Akt and IRS1 in the WAT of obese UCP1-KO mice. Western blot analysis for mTOR (A), Akt (B) and IRS1 (C) was done with tissue lysates (50 µg protein) of epididymal WAT from the UCP1-KO mice fed a high-fat diet with or without evodiamine for 2 months as described in Supplemental Methods. Phosphorylation levels of mTOR Ser2448, Akt Ser473 and IRS1 Ser636/639 were normalized to the total level of each protein. Data are expressed as mean±SEM (*n* = 4). **p* <0.05 *vs*. control group.

Figure S3.

Effect of evodiamine on phosphorylation of mTOR and UCP1 in the BAT of KK-Ay mice treated with evodiamine. Western blot analysis for mTOR (A) and UCP1 (B) was done with tissue lysates (30 µg protein) of BAT from KK-Ay mice treated with evodiamine for 7 days. Levels of mTOR Ser2448 phosphorylation and UCP1 protein were normalized to the total mTOR and tubulin levels, respectively. Data are expressed as mean±SEM (*n* = 5).

Figure S4.

Effect of evodiamine on phosphorylation of mTOR, S6K, Akt and IRS1 in the gastrocnemius muscle (GM) of KK-Ay mice treated with evodiamine. Western blot analysis for mTOR (A), S6K (B), Akt (C) and IRS1 (D) was done with tissue lysates (30 µg protein) of GM from KK-Ay mice treated with evodiamine for 7 days. Phosphorylation levels of mTOR Ser2448, S6K Thr389, Akt Ser473 and IRS1 Ser636/639 were normalized to the total level of each protein. Data are expressed as mean±SEM (*n* = 5).

Figure S5.

Effect of evodiamine on phosphorylation of ERK, Akt and IRS1 in pre-adipocytes. 3T3-L1 pre-adipocytes were serum-deprived for 4 h and then treated with 20 µM evodiamine for 1 h and with 20 nM insulin for the last 10 min. Western blot analysis for ERK, Akt and IRS1 were done with cell lysates (30 µg protein).

Figure S6.

Measurement of oxygen consumption (VO_2_) and respiratory quotient (RQ) in UCP1-KO mice with or without treatment with evodiamine. VO_2_ (A) and RQ (B) were measured for 24 h in male mice injected with vehicle or evodiamine (3 mg/kg BW) at 14:00 h. C**,** Data on RQs were analyzed as relative cumulative frequency (PRCF). Each curve represents 1365 measurements of RQ from three mice for each group as described in Supplemental Methods. Data are expressed as mean±SEM (*n* = 3 for each group). ****p* <0.001 *vs*. vehicle group.

Figure S1.

Figure S2.

Figure S3.

Figure S4.

Figure S5.

Figure S6.


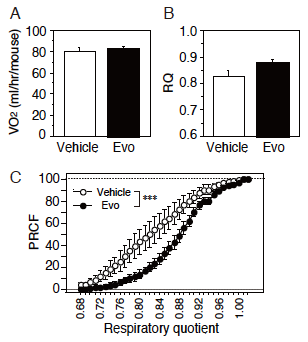

Supplement: File S1 — Methods S1, Animal experiments using UCP1-knockout (KO) mice and cell culture experiments using 3T3-L1 pre-adipocytes. References S1, References for supplementary methods. Figure S1, Effect of evodiamine on mTOR-S6K signaling in the RWAT of KK-Ay mice treated with evodiamine. Western blot analysis of mTOR (A), S6K (B), Akt (C) and IRS1 (D) was done with tissue lysates (30 µg protein) of retroperitoneal WAT from KK-Ay mice treated with evodiamine for 7 days. Phosphorylation levels of mTOR Ser2448, S6K Thr389, Akt Ser473 and IRS1 Ser636/639 were normalized to the total level of each protein. Data are expressed as mean±SEM (n = 5). *p<0.05 vs. vehicle group. Figure S2, Effect of evodiamine on phosphorylation of mTOR, Akt and IRS1 in the WAT of obese UCP1-KO mice. Western blot analysis for mTOR (A), Akt (B) and IRS1 (C) was done with tissue lysates (50 µg protein) of epididymal WAT from the UCP1-KO mice fed a high-fat diet with or without evodiamine for 2 months as described in Supplemental Methods. Phosphorylation levels of mTOR Ser2448, Akt Ser473 and IRS1 Ser636/639 were normalized to the total level of each protein. Data are expressed as mean±SEM (n = 4). *p<0.05 vs. control group. Figure S3, Effect of evodiamine on phosphorylation of mTOR and UCP1 in the BAT of KK-Ay mice treated with evodiamine. Western blot analysis for mTOR (A) and UCP1 (B) was done with tissue lysates (30 µg protein) of BAT from KK-Ay mice treated with evodiamine for 7 days. Levels of mTOR Ser2448 phosphorylation and UCP1 protein were normalized to the total mTOR and tubulin levels, respectively. Data are expressed as mean±SEM (n = 5). Figure S4, Effect of evodiamine on phosphorylation of mTOR, S6K, Akt and IRS1 in the gastrocnemius muscle (GM) of KK-Ay mice treated with evodiamine. Western blot analysis for mTOR (A), S6K (B), Akt (C) and IRS1 (D) was done with tissue lysates (30 µg protein) of GM from KK-Ay mice treated with evodiamine for 7 days. Phosphorylation levels of mTOR Ser2448, S6K Th [file pone.0083264.s001.docx]
